# Thickness and yttria percentage influences the fracture resistance of laminate veneer zirconia restorations

**DOI:** 10.1002/cre2.658

**Published:** 2022-09-13

**Authors:** Ali Dhahee Malallah, Nadia H. Hasan

**Affiliations:** ^1^ Department of Conservative Dentistry, College of Dentistry University of Mosul Mosul Iraq

**Keywords:** laminate veneers, thickness, yttria percentage, zirconia ceramic

## Abstract

**Objectives:**

Mechanical properties are cardinal for the long‐term clinical success of laminate veneer restorations but the selection of new restorative materials should ideally be based on clinical evidence, therefore, in vitro testing of dental materials is a good alternative to evaluate their properties and understand their behavior so this study aimed to compare and evaluate the effect of two different thicknesses and yttria percentage on the fracture resistance of laminate veneer zirconia restorations.

**Materials and Methods:**

Forty laminate veneer restoration prepared from partial sintering zirconia of 3Y (yttria), 5Y (yttria), combined 3Y&5Y (yttria), and lithium disilicate. Specimens were assigned into four main groups according to their percentage of yttria content (*n* = 10) and subgrouped into two thicknesses (0.5 mm thickness and 0.3 mm thickness) (*n* = 5) as follows: Group I, II, III, and IV (Group I for lithium disilicate (control), Group II for 3Y zirconia, Group III for 5Y zirconia, and Group IV for combined 3Y&5Y zirconia), each of them subdivided according to their thickness into two subgroups (*n* = 5 for each one) and resistance to fracture for each restoration was evaluated using a universal testing machine. Data were analyzed using a one‐way analysis of variance and Duncan's tests at a 5% level of significance.

**Results:**

The thickness of laminate veneer restoration significantly affects the fracture resistance value of all type of laminate veneers restorations (fracture resistance mean value was highest for 0.5 mm thickness and lower for 0.3 mm thickness restorations) and yttria percentage significantly affect fracture resistance value of zirconia laminate veneer restorations (fracture resistance mean value was highest for 0.5 mm thickness of 3Y zirconia [865 N] and combined 3Y&5Y zirconia [846 N]).

**Conclusions:**

Reducing the thickness of laminate zirconia veneer restorations to 0.3 mm reduces its fracture resistance and increasing yttria percentage had an adverse effect on fracture resistance of zirconia laminate veneer restorations.

## 1 INTRODUCTION

Personal appearance is getting more and more important in society as it is the aesthetic aspect that is primarily realized in people. Relative to crowns, porcelain laminate veneers (PLVs) is a conservative treatment option to improve anterior aesthetics and have a long history of documented success. Many types of veneer preparation models have been recommended in the literature; however, two of them are more commonly used because they provide more benefits. These are (1) the “feather edge preparation,” in which the labial surface is minimally prepared up to the incisal edge and (2) the “incisal overlap preparation,” in which the incisal edge is reduced (Chai et al., 2020; Duzyol et al., 2016a).

For adhesive cementation, resin‐based cements are preferred. Chemical, light, or dual‐cure polymerizations were all options. Conventional cements, such as resin‐modified glass ionomer and zinc phosphate, can still be used with caution if the abutment preparation ensures sufficient retention and it had been shown that the traditional cements were more commonly associated with loss of retention of fixed prostheses than those cemented with resin cements (Le et al., 2015).

Resin cements (light‐cured only) have the benefit of providing a longer working period and curing on demand, as well as a consistent color enhancement. They are mostly used to cement translucent thin ceramic restorations like laminate veneers (Mendonca et al., 2019).

Lithium disilicate is a type of ceramic material commonly used for veneer construction, which is made up of a glass matrix with scattered crystalline minerals and has good optical properties. However, as opposed to other ceramic materials, these systems have lower mechanical properties, including fracture toughness and flexural strength (Manziuc et al., 2019).

Zirconia‐based ceramics outperform lithium disilicate ceramics in terms of mechanical efficiency, strength, and fracture resistance. The mechanical and optical properties of zirconia vary depending on the mol concentration of yttria. 3Y‐partially stabilized zirconia (PSZ) has the highest mechanical properties due to the high tetragonal particles that lead to transformation toughening, which prevents crack propagation (Michailova et al., 2020).

To improve its translucency increasing the percentage of yttria within the Y‐PSZ up to 5% production for 5Y‐PSZ with large grain size and the replacement of tetragonal grains with cubic grains leads to a light‐scattering and birefringence reduction at grain boundaries with an ultratranslucency (Zhang & Lawn, 2018)

To enhance the strength combination of 3Y‐ and 5Y‐PSZ developed by combining two generations of zirconia (different percentages of yttria) in one blank to obtain the benefits of both zirconia generations. This is primarily a combination of a high‐flexural‐strength 3Y‐TZP in the dentin/body region with a high‐translucency 5Y‐TZP in the incisal or occlusal areas for improved esthetics (Michailova et al., 2020).

The null hypothesis proposed that there is no difference in fracture resistance between different thicknesses of laminate veneers with different percentages of yttria for zirconia laminates veneer restorations in this study.

So the aim of this study was to compare and evaluate the effect of two different thicknesses and yttria percentage on the fracture resistance of laminate veneer zirconia restorations.

## 2 MATERIALS AND METHODS

Four ceramic materials used in this study (IPS e.max ZirCADprime, which represented the combined 3Y&5Y zirconia from Ivoclar Vivadent; Schaan, Liechtenstein, DD cubeX2 ML, which represented the 5Y zirconia from Dental Direkt; Industeriezentrum, Spenge‐German, copra supreme symphony, which represented the 3Y zirconia from Whitepeaks), and lithium disilicate IPS e.max CAD blocks from Ivoclar Vivadent. Specimens were assigned into four main groups according to their percentage of yttria content.

**Figure 1:**
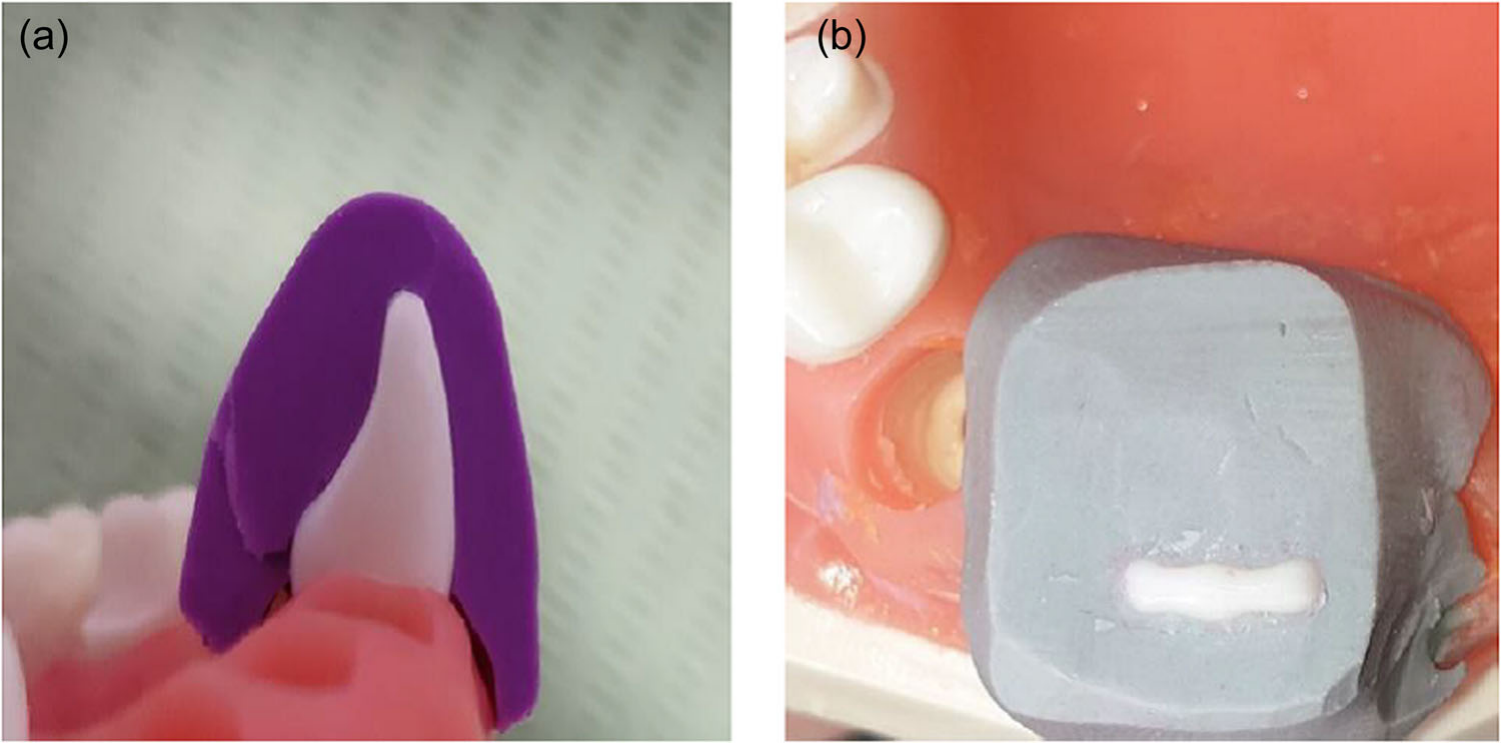
Silicone index preparation: (a) side view of the silicone index (b) incisal view of the silicone index.

**Figure 2:**
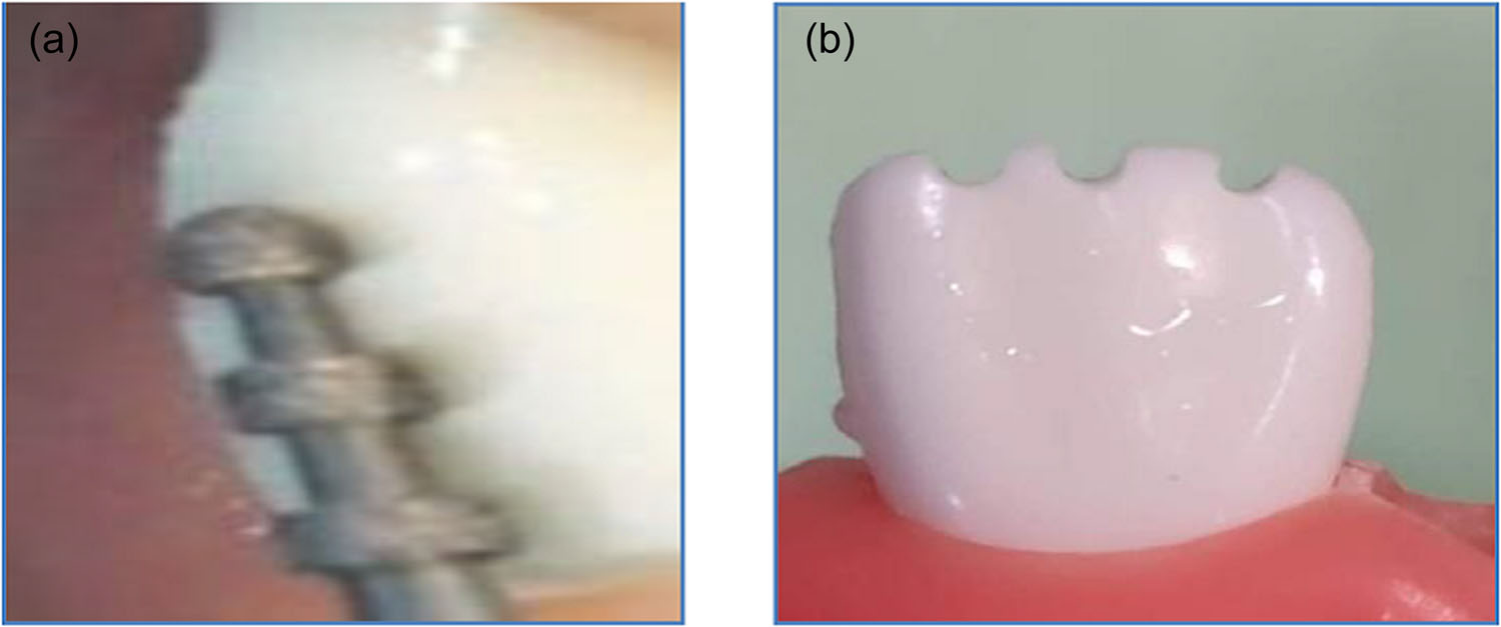
Standardization of the reductions (a) guiding grooves created on the facial surfaces (b) Incisal reduction.

**Figure 3:**
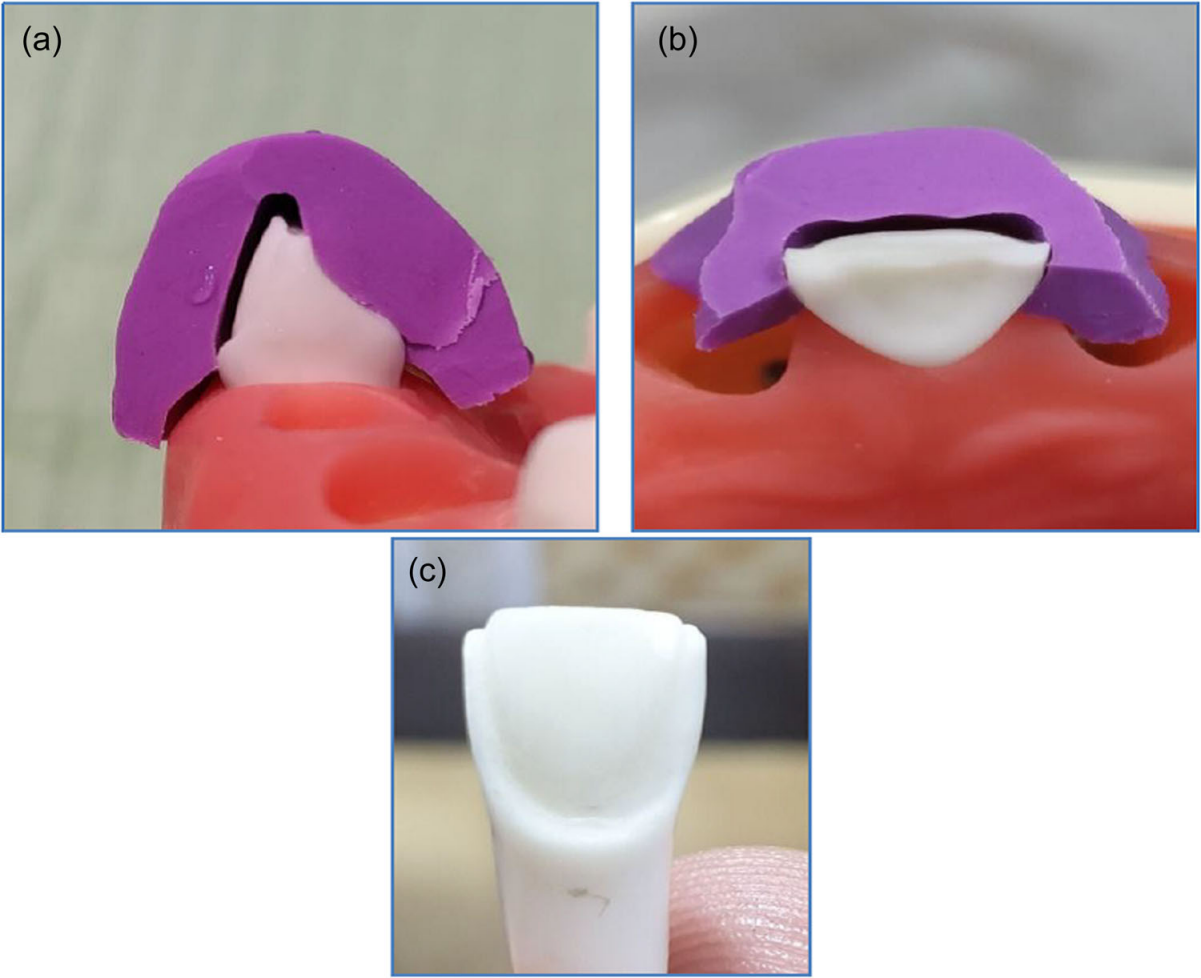
Veneer preparation with silicone index: (a) side view of the preparation; (b) incisal view of the preparation; (c) completely prepared plastic tooth.

Two plastic teeth have been selected for preparing laminate veneer restorations (0.3 and 0.5 mm thicknesses). To obtain standardization for the preparation, heavy body rubber base silicone impression material as a silicone index was prepared, as shown in Figure 1a,b. An incisal overlap design for veneer prepared on plastic teeth with two different preparation depths labially by using self‐limiting depth‐cutting burs of 0.5 and 0.3 mm, while the reduction for the incisal area was 1.5 mm with palatal overlap for 1.0 mm below the reduction with chamfer finishing line gingivally (1 mm) above the cement enamel junction (CEJ) (Chai et al., 2020); the amount of incisal reduction was calculated as 1.5 mm with the help of the diameter of the diamond bur by using veneer preparation kit (DiaTessin), as shown in Figures 2a,b and 3a–c.

The prepared plastic teeth were then scanned digitally for making (40) nickel chromium dies to overcome some of the drawbacks of natural teeth, such as differences in size, form, and individual structure caused by the patient's age, location, and duration for storing extracted tooth, and the effect of the adhesive bond strength on the biomechanical behavior of the laminate veneer; the test was conducted on nickel–chromium abutments to standardize samples (Zlatanovska et al., 2019) that were then finished, oxidized, and sandblasted to enhance cementation of the laminate veneers. The metal die was scanned to construct 40 laminate veneers (10 veneers for each ceramic material) (Zircad Prime, DD cubeX2 ML, CopraSupreme Symphony, and lithium disilicate material IPS e.max CAD as a control group).

### 2.1 Design of the study

**Figure 4:**
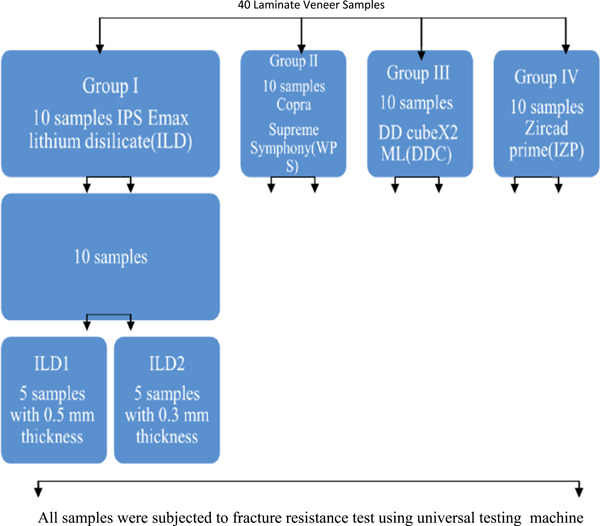
Experimental design of the fracture strength test for zirconia and lithium disilicate samples.

The samples that have been prepared in this study were divided into four main groups according to the type of the material (Ivoclar Zircad Prime, DD cubeX2 ML, CopraSupreme Symphony, and Ivoclar lithium disilicate IPS e.max CAD material) and each of them subgrouped into two thickness ILD1 (0.5 mm Ivoclar lithium disilicate material IPS e.max CAD), ILD2 (0.3 mm Ivoclar lithium disilicate material IPS e.max CAD), DDC1 (0.5 mm Dental direct cubeX2 ML), DDC2 (0.3 mm Dental direct cubeX2 ML), WPS1 (0.5 mm whitepeaks copra supreme symphony), WPS2 (0.3 mm white peaks CopraSupreme Symphony), IZP1 (0.5 mm Ivoclar Zircad Prime), and IZP2 (0.3 mm Ivoclar Zircad Prime) as shown in Figure 4.

### 2.2 Preparing the samples for testing

An autopolymerized acrylic resin material was used as a base to fix each metal die 1 mm apical to the CEJ to simulate the natural biologic width and the base was held in a metallic mold (Chai et al., 2020; Gupta et al., 2018; Tomer et al., 2018).

### 2.3 Cementation of the laminate veneers

Before cementation, each laminate veneer was checked for fitness and thickness, and then conditioning of veneers and their corresponding metal dies were performed as follows:

A. Conditioning of the E‐max laminate veneers: done by etching with hydrofluoric acid (ceramic etching gel; Ivoclar‐Vivadent) for 20 s, rinsed and ultrasonic cleaning for 5 min, then air‐dried and treated with a primer left for 60 s, and air‐dried for 5 s (Monobond plus primer Ivoclar Vivadent) (Gresnigt et al., 2021; Linhares et al., 2019).
B. Conditioning of zirconia‐based laminate veneers: through sandblasting for 10 s at 0.2 MPa pressure using 50 μm diameter Al_2_O_3_ particles, then ultrasonic cleaning for 5 min and dried, then primer left for 60 s and air dried for 5 s (Monobond plus primer Ivoclar Vivadent) (Gresnigt et al., 2021; Saker & Özcan, 2021).
C. Conditioning of the corresponding metal dies surfaces: done by oxidation, sandblasting for 20 s at 0.3 MPa pressure using 150 μm diameter Al_2_O_3_ particles, ultrasonic clean for 5 min, air dried and finally primer applicated for 60 s and air dried for 5 s (Kapoor et al., 2017).

Then a dual cure resin cement (VariolinkEsthetic DC Ivoclar Vivadent) was applied on the intaglio surface of laminate veneer to ensure complete curing of the cement and it was used after monobond plus prime being used for conditioning of the intaglio surface of the restorations) then seated on their corresponding metal die and allow to set under a constant load of 1 kg for 20 s using a special customized cementing device and light polymerization was done on each surface for 40 s (Elgamma et al., 2018).

### 2.4 Testing of the samples

Each sample will be tested individually using the electronic universal testing machine (Gester universal testing machine GT‐UA03). A compressive mode of the load is applied at 135° angle, which simulates the force that laminate veneers are subjected to inside the mouth (Alghazzawi et al., 2012; Tsouknidas et al., 2020). To create 135° angle, the metal base for holding the sample at 45° angle was fixed in palce within the prepared mounting jig. Securing the sample in its place within the prepared metal base and a load applied using a metallic rod with a flat end circular surface (4 mm diameter) attached to the upper movable compartment of the electronic universal testing machine traveling at a cross‐head speed of 1 mm/min that will touch the incisal edge at 135° (Alghazzawi et al., 2012; Blunck et al., 2020; Duzyol et al., 2016) as shown in Figure 5, 6. Failure will be defined as the occurrence of load drops and acoustic events and the peak value will be stored in the computer software and recorded in Newton. All the recorded data will be collected and tabulated to be statistically analyzed.

**Figuree 5:**
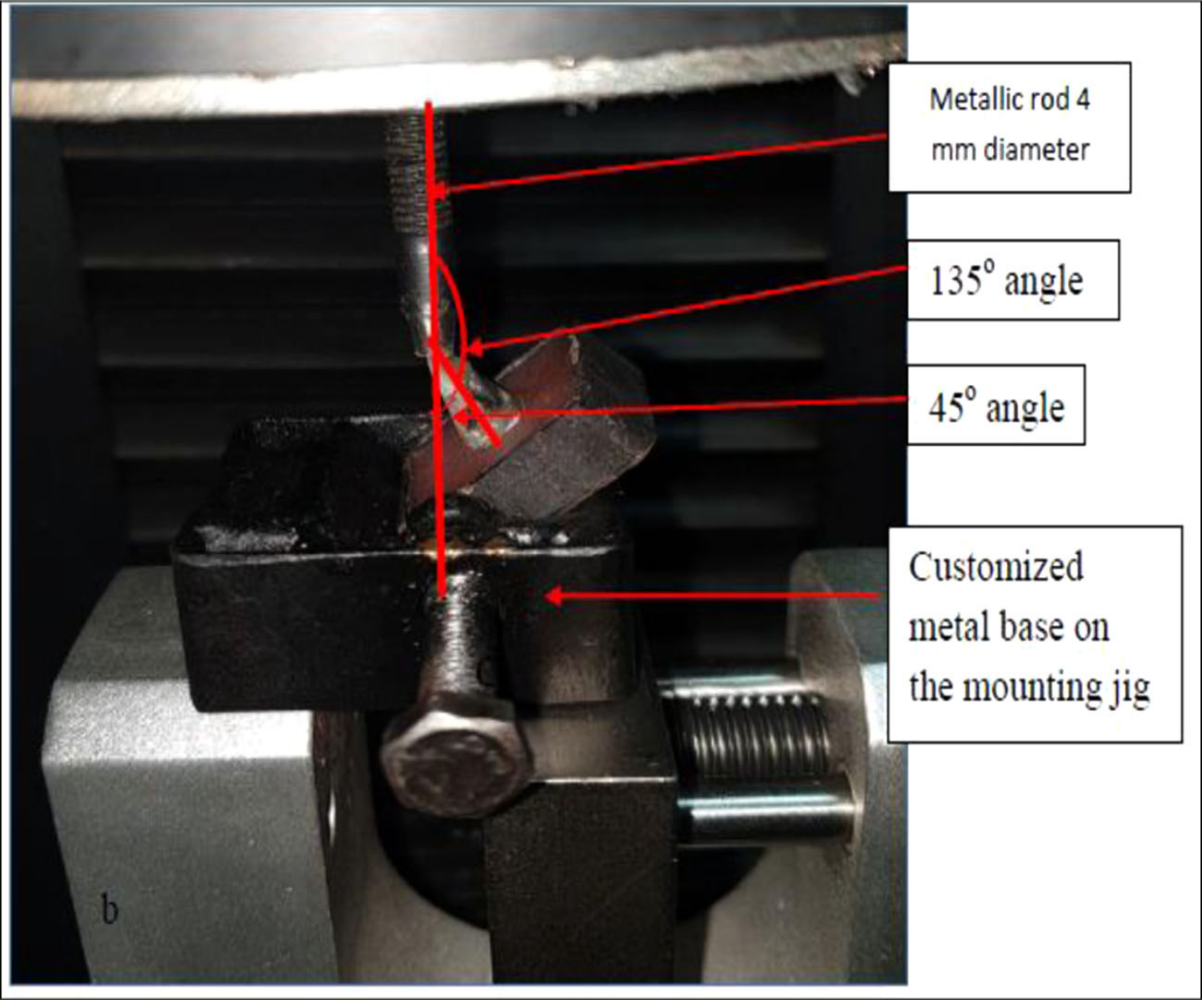
Testing of the samples by Gester universal testing machine (GT UA03) using a customized metal base for holding the sample at 45° angle and a metallic rod at 135° to the incisal edge.

### 2.5 Statistical analysis

Data were analyzed by using SPSS (statistical package of social science) software version 26.0 Statistics:

1. Normality of data distribution: by using Kolmogorov–Smirnov and Shapiro–Wilk tests, to detect data distribution and apply the proper statistical test.
2. Mean, standard deviation (SD), minimum and maximum value of fracture resistance (descriptive statistics).
3. Duncan's multiple range test was used to evaluate the effect of thickness and yttria percentage on fracture resistance of laminate veneers with the level of significance that was used (.05 ≥ *p*) (this paragraph is added to Section 2).

## 3 RESULTS

Descriptive statistics including the mean and SD for the different types of ceramics materials and their effect on fracture resistance of zirconia and lithium disilicate as shown in Table 1.

**Table 1:** Descriptive statistics for different types of ceramics materials and their effect on fracture resistance.

	*N*	Mean	Std. deviation	Std. error	Minimum	Maximum
ILD1	5	410.1	18.5	8.4	381.7	432.3
ILD2	5	331.9	33.5	14.9	296.6	378.2
DDC1	5	727.5	37.5	16.8	667.8	765.4
DDC2	5	478.5	48.9	21.9	423.5	539.7
WPS1	5	865.0	45.3	20.3	811.9	920.1
WPS2	5	518.8	67.5	30.2	413.7	592.9
IZP1	5	846.0	78.3	35.0	768.1	974.4
IZP2	5	539.8	32.6	14.6	496.2	574.8

*Note*: ILD1 (0.5 mm Ivoclar lithium disilicate material IPS e.max CAD), ILD2 (0.3 mm Ivoclar lithium disilicate material IPS e.max CAD), DDC1 (0.5 mm Dental direct cubeX2 ML), DDC2 (0.3 mm Dental direct cubeX2 ML), WPS1 (0.5 mm white peaks copra supreme symphony), WPS2 (0.3 mm white peaks CopraSupreme Symphony), IZP1 (0.5 mm Ivoclar Zircad Prime), and IZP2 (0.3 mm Ivoclar Zircad Prime).

Regarding fracture resistance of the different zirconia ceramics of different yttria percentages with two thicknesses and lithium disilicate ceramics with two thicknesses, one‐way analysis of variance showed that there was a significant difference between ceramic materials at (*p* ≤ .05), as shown in Table 2.

**Table 2:** ANOVA (comparison between different ceramic veneer materials)

	Sum of squares	*df*	Mean square	*F*	Sig.
Between groups	1,161,851.100	7	165,978.729	52.185	0.000
Within groups	101,778.476	32	3180.577		
Total	1,263,629.576	39			

Abbreviation: ANOVA, analysis of variance.

**Figure 6:**
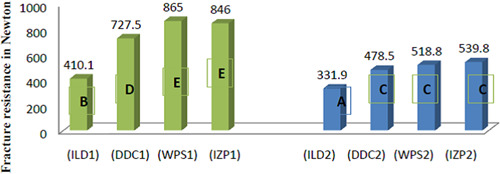
Column graph for Duncan's multiple range test at a 5% significance level that showed the effect of different yttria percentages and two thicknesses on the fracture resistance value.

To determine the level of significance that was obtained, Duncan's post hoc test at a 5% level of significance showed the fracture resistance value of IZP1 (0.5 mm Ivoclar Zircad Prime) and WPS1 (0.5 mm white peaks copra supreme) groups were significantly higher than all other groups (*p* ≤ .05), as shown in Figure 5, 6.

## 4 DISCUSSION

Natural teeth were avoided as they might have invisible cracks or inconsistent dentine structure that may cause the tooth to fracture at high loads during testing and cause some restrictions in the reproducibility and comparability between natural teeth specimens (Abdel‐Nabi et al., 2019; Zlatanovska et al., 2019).

The test was conducted on nickel–chromium abutments to standardize samples to overcome some of the drawbacks of natural teeth, such as differences in size, form, and individual structure caused by the patient's age, the location and duration for storing extracted tooth, and the effect of the adhesive bond strength on the biomechanical behavior of the laminate veneer (Zlatanovska et al., 2019).

### 4.1 The effect of two thicknesses on fracture resistance of laminate veneers

Today, minimally invasive restorations with a high‐level‐esthetic appearance have become available for dental restorations, mainly because of the introduction of oxide‐based ceramics and it has been stated that the small particle size of zirconia makes it possible to produce thin ceramic dental restorations. However, the quality of zirconia can be affected, for example, by the grain size, type of stabilizing oxide, heat treatment, manufacturing process, and composition (Lucas et al., 2015; Sundh et al., 2019).

Tooth preparation for laminate veneer is crucial for optimal function and esthetics; therefore, care should be taken to completely perform this preparation in the enamel. In general, anterior tooth preparation requires a 0.3‐ to 0.7‐mm facial reduction, depending on the location of the crown, to imitate the natural contours of the tooth. This anatomical preparation technique may enable tooth preparation within only enamel because enamel thickness is different in different zones of the tooth (Oztürk & Bolay, 2014).

One of the main objectives of this study was to evaluate the fracture resistance of laminate veneers fabricated with two different preparation depths. Ferrari et al. (1992) reported the thickness of the enamel layer for anterior teeth and showed that the central incisors have 0.3–0.5 mm enamel on the cervical part, and 0.6–1.0 mm enamel on the middle, and 1.0–2.1 mm on the incisal part. The reduction in measurements of the preparations in enamel and dentin found in the present study parallels this study. The quantity of incisal reduction is determined by considering the portion recommended for esthetics as 1.5–2 mm in anterior laminate veneer restorations (Ferrari et al., 1992).

In the present study, the fracture strength of all types of ceramics materials had a significant difference in fracture resistance between the two veneer thicknesses (0.5 and 0.3 mm) where it was as follows: for 0.5 mm were 410.1, 727.5, 865, and 846 N for ILD1, DDC1, WPS1, and IZP1, respectively, where it was highest for WPS1 and IZP1 groups as shown in Figure 5, 6 and this finding was in consistent with other researchers whom concluded that reducing veneer thickness decreased its fracture strength that compare 0.5 and 0.7 mm and 1 mm thicknesses and using veneer made of nonpolycrystalline ceramics (Alraheam et al., 2020; André et al., 2016).

While other studies found that reducing thickness exhibited superior fracture strength or no significant difference and it was proposed that the monoclinic phase/tetragonal phase distribution in the Y‐TZP could have influenced the outcome or they explained that by the difference in the methodology or one possible explanation for thinner Y‐TZP ceramics, resulting in higher strength could be the phase transformation t‐m created on the surface because of the machining process and a proportionally thicker monoclinic layer may be created on thinner specimens than on thicker specimens so Since the monoclinic layer has; 3%–4% volume expansion compared to the tetragonal phase, the compressive layer formed at the surface by this layer could have resulted in stronger thinner specimens (Akesson et al., 2009; Baptista da Silva et al., 2020; Sundh & Sjögren, 2004).

So the null hypothesis was rejected as a significant difference was found in fracture resistance between two different laminate veneer thicknesses (0.5 and 0.3 mm).

WPS1 and IZP1 groups (865 and 846 N), respectively, had the highest fracture load among all groups at different thicknesses as shown in Figure 5, 6, this may be related to the contents of 3Y % of yttria and the increased amount of tetragonal phase, which lead to transformation toughening where the transition from tetragonal to monoclinic associated with 3%–4% volume expansion followed by compressive stress that opposes the crack tip and restricts it from propagating, causes fractures and flaws to be arrested (Tanaka et al., 2019). These  findings were in consistent with results of studies that found increasing yttria percentage had adverse effect on zirconia fracture strength (Alraheam et al., 2020; Elsayed et al., 2019; Michailova et al., 2020; Skjold et al., 2020; Yan et al., 2018).

The mechanical resistance of the veneer depends on the type and the shape of the preparation, which altogether can resist the occlusal and lateral forces of the chewing pressure during mastication. Before the preparation, it is very important to decide whether the incisal edge will be reduced. Until now, there are still insufficient data regarding the best type of veneer preparation. Very few studies have focused on the impact of the preparation design on the success and durability of the restoration (Zlatanovska et al., 2019).

So, the tested null hypothesis that the investigated ceramics result in comparable mechanical strength was rejected.

## 5 CONCLUSION

Within the limitation of this study, reducing the thickness of laminate veneer restorations reduce its fracture resistance and also increasing yttria percentage had an adverse effect on fracture resistance of zirconia laminate veneer restorations but in both situation, they still within the physiologic biting forces (anterior teeth) in adults which are between 108 and 230 N which suggests use of zirconia as laminate veneer material with reduced thickness and high yttria percentage.

## CONFLICT OF INTEREST

The authors declare no conflict of interest.

## ETHICS STATEMENT

The present study has no human or animal participation and the study was performed on lab models, hence informed consent was not required.

## Data Availability

The data that support the findings of this study are openly available in [repository name, e.g., “figshare”] at [doi], reference number [reference number].
